# Primary Cutaneous Adiaspiromycosis:A Case Report and Review of the Literature

**DOI:** 10.5146/tjpath.2025.13977

**Published:** 2026-09-15

**Authors:** Sateesh Chavan s, Chandrashekar T N, Arathi S, Sunitha Vernekar

**Affiliations:** Department of Pathology, Karnataka Medical College & Research Institute (KMC RI), Karnataka, İndia; Department of Pathology, Shivamogga Institute of Medical Sciences, Karnataka, İndia

**Keywords:** Adiaspiromycosis, Haplomycosis, Chrysosporium parvum var crescens, Emmonsia crescens, Adiaspirosis

## Abstract

Aim: To document a case of primary cutaneous Adiaspiromycosis.

Introduction: Adiaspiromycosis is a rare emerging fungal infection caused by Chrysosporium Parvum var Crescens now called as “Emmonsia Crescens” belonging to the genus Emmonsia, order Onygenales, family Ajellomycetes.

Case Report: A 38-year-old male presented with an ulcerated nodule over the infra-axillary region with itching for 3 months. The lesion began as small nodule slowly growing to attain the present size. His routine investigations were within normal limits. Serological tests such as HBsAg and HIV were nonreactive. Fine needle aspiration cytology showed ‘suppurative granulomatous’ inflammation. The nodule was excised and sent for histopathological examination. Histopathology showed numerous noncaseating granulomas and suppuration. Amidst suppuration, as well as inside the giant cells and within granulomas, numerous varying sized, thick-walled, round to oval ‘adiaspores’ were seen. There was no evidence of budding or septation or endosporulation. They were PAS and GMS positive. He was treated with topical luliconazole and oral fluconazole. There was no recurrence on follow up of one years.

Conclusion: Adiaspiromycosis is a rare fungal infection and primary cutaneous involvement is a rare distinct entity. Detailed morphological assessment in histopathology is essential for its identification as the organisms are difficult to isolate in fungal culture from human clinical material.

## Introduction

Adiaspiromycosis (or Adiaspirosis or Haplomycosis) is a rare emerging fungal infection caused by Chrysosporium Parvum var Crescens (Emmonsia Crescens). The disease is more common amongst primates and domestic animals and is rare in humans. Humans are accidental hosts and dead end of life cycle for the fungi. Humans contract the infection either by inhalation or inoculation of ‘spores.’ The lungs are the most common site affected but other sites can be involved. They usually remain localized to the site of entry, which only grow in size without actual division, to form large sized spores called ‘adiaspores’ that elicit suppurative granulomatous inflammation and form a space-occupying lesion called ‘Adiaspiroma’. Dissemination is rare. Histopathology is the mainstay in diagnosis. Often the lesion is an incidental finding during histopathological examination of tissue. The organisms are difficult to isolate from human clinical material (1-5).

Primary cutaneous involvement is a rare distinctive entity. Here we are presenting a case report of cutaneous involvement of adiaspiromycosis.

## Case history

A 38-year-old male timber yard worker presented with an ulcerated nodule over the infraaxillary region with itching for 3 months. The lesion began as small nodule slowly growing to attain the present size. There was no history of fever, trauma, diabetes and hypertension or tuberculosis. On examination, there was a subcutaneous ulcerated nodule over the left infraaxillary region measuring 2 cm x 1 cm x 0.8 cm, freely mobile and slightly tender. His cardiovascular, respiratory and central nervous system functions appeared normal. His chest x ray was unremarkable. His routine investigations revealed hemoglobin of 13.88 gm%, total leukocyte count of 8.340 x 103/μL, platelet count of 320 x 103/μL, and the differential count showed 49% polymorphs, 45% lymphocytes, 5% eosinophils, and 1% monocyte. His random blood sugar was 118 mg% and serological tests such as HBsAg and HIV were nonreactive. Fine needle aspiration cytology showed a ‘suppurative granulomatous’ inflammation. The nodule was excised and sent for histopathological examination.

**Figure 1:**
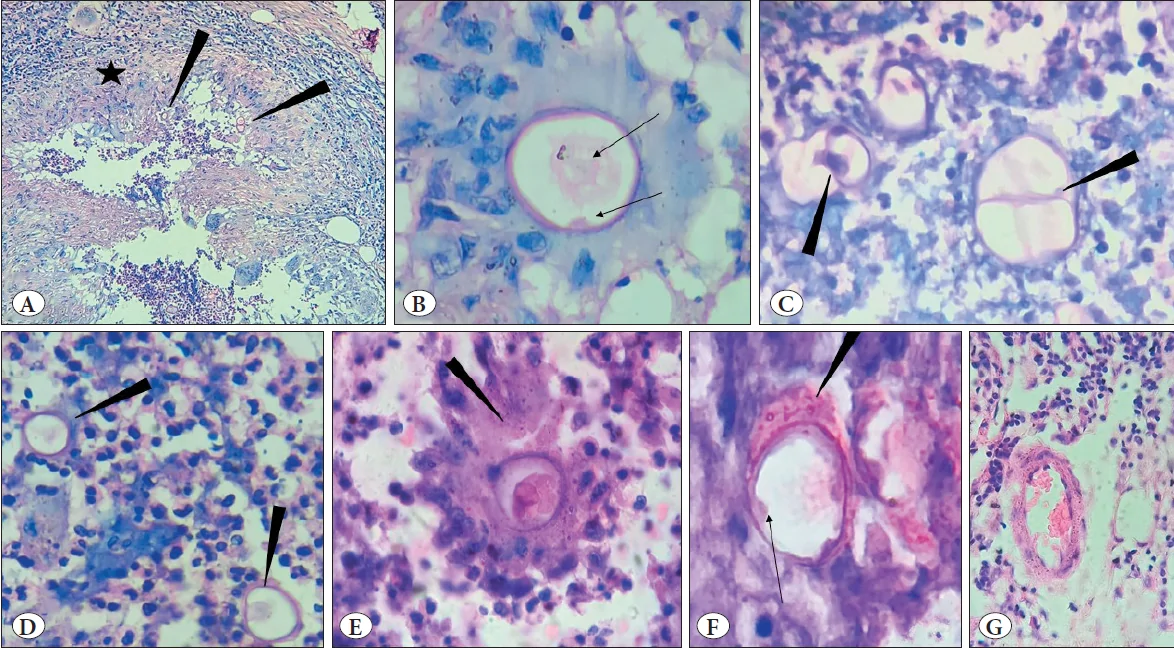


Routinely processed Hematoxylin & Eosin-stained paraffin sections showed numerous discrete nodular areas interspersed with suppuration and fibrocollagenous and fibroadipose tissue. Nodular areas showed discrete well-delineated epithelioid cell granulomas with both foreign body and Langhans type giant cells. Most granulomas were nonnecrotic; however some were disrupted by central suppuration and some coalesced to form larger granulomas (Figure 1A).

**Figure 2:**
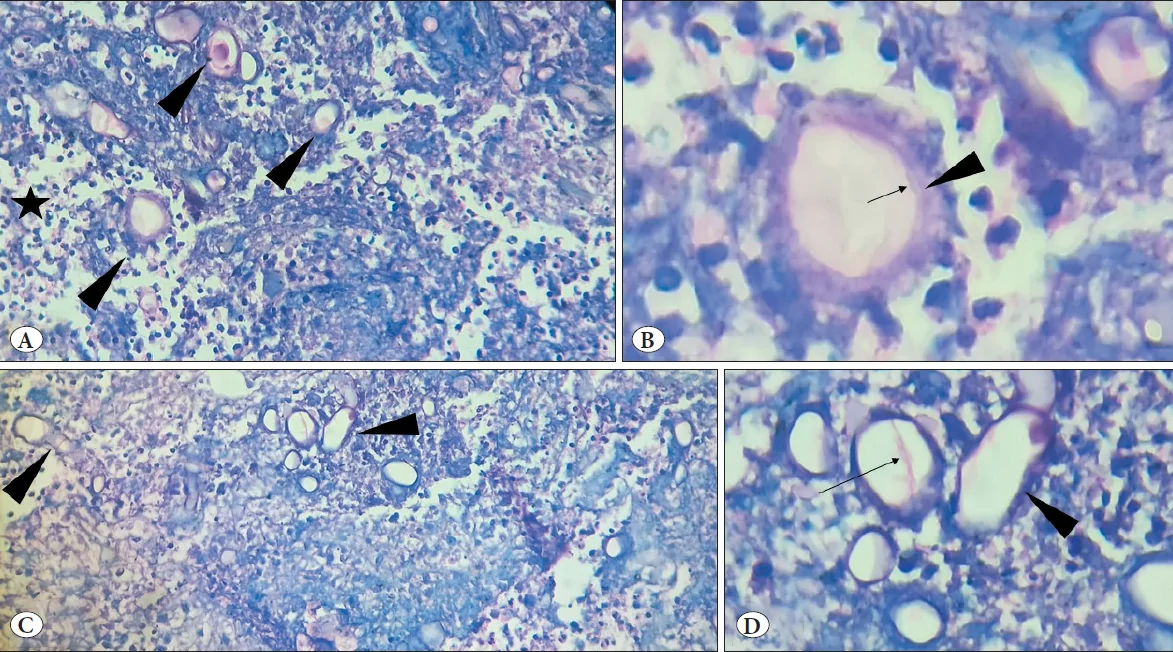
Showing numerous thick walled adiaspores (black arrow heads) amidst suppuration (Asterix) (x 40, H&E), B) Inset of A: trilaminar thick wall of adiaspore showing outer thick eosinophilic zone (black long arrow head), wider pale pink middle zone and inner pale irregular zone (black arrow) (x200, H&E). C) elongated adiaspore simulating ‘hyphae’ (black arrow heads) (x100, H&E), D) inset of C. adiaspore showing retraction of inner wall (black arrow) (x200, H&E).

**Figure 3:**
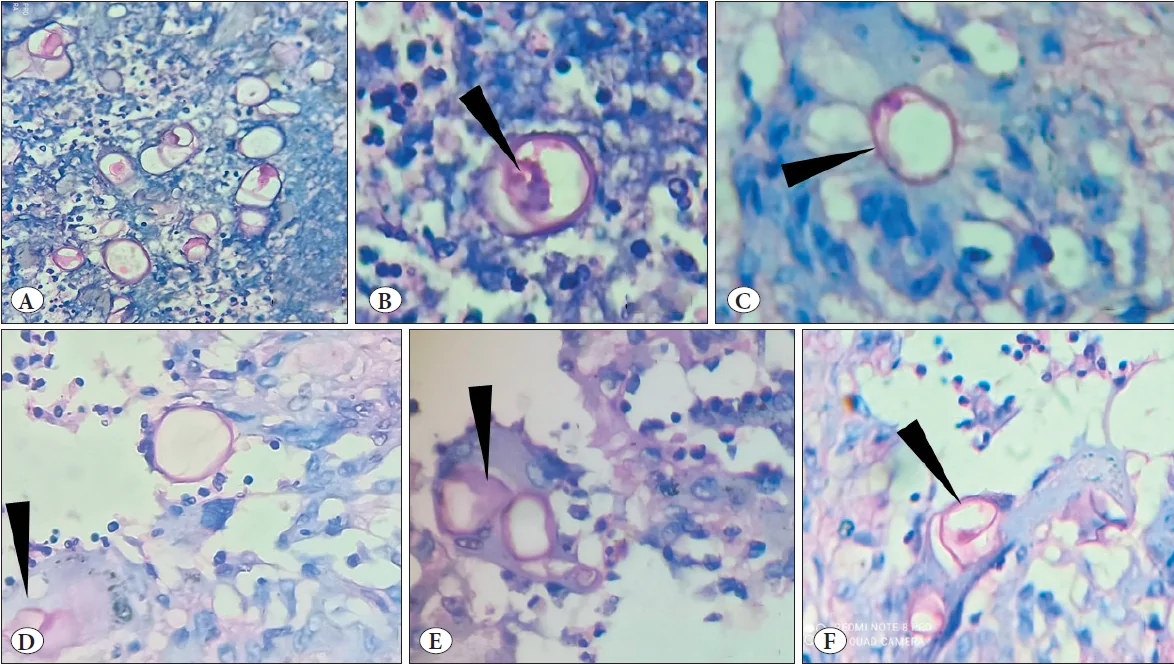
Showing Periodic acid Schiff positive ‘adiaspores’. A) ‘adiaspores’ amidst suppuration (x40, PAS), B) adiaspore with retraction of inner wall on one side (black arrow head) with adherent irregular amorphous greyish pink granular material (x100, PAS), C) ‘adiaspore’ amidst granuloma (black arrow head) (x100, PAS), D,E) ‘adiaspore’ inside multinucleated giant cell (black arrow heads) (x40, PAS), F) degenerating & collapsed adiaspore inside a multinucleated giant cell (black arrow head) (x 40, PAS).

**Figure 4:**
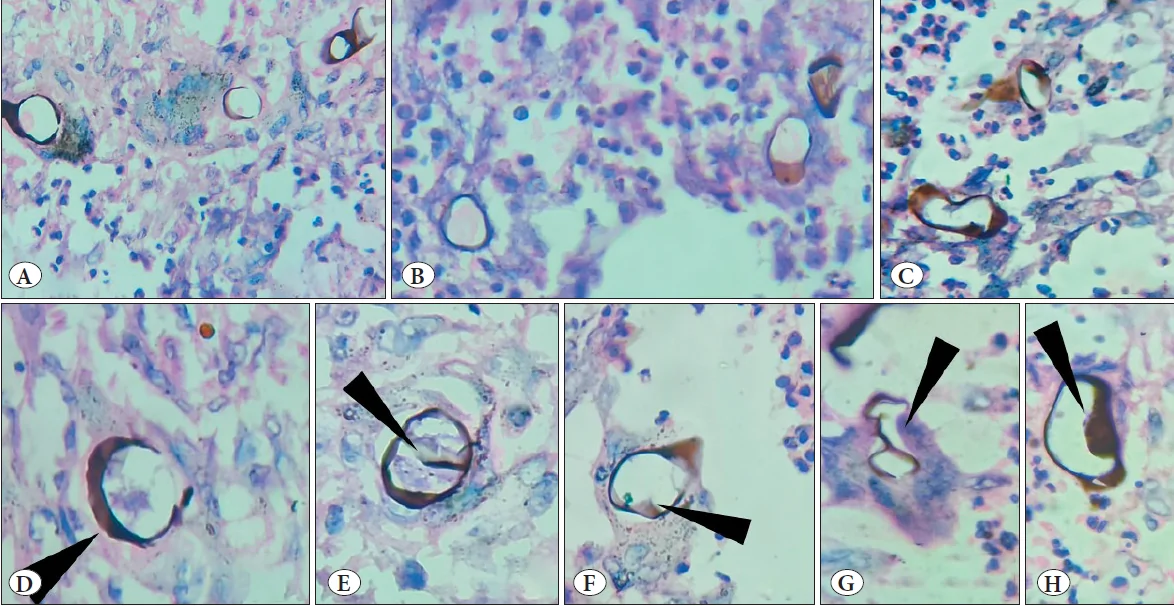
Showing Gomori’s methenamine silver stain positive adiaspores. A) varying sized ‘adiaspores’ amidst granulomas (x40, GMSHE), B,C) ‘adiaspores’ amidst suppuration (x40, GMS-HE), D) ‘adiaspore’ inside a multinucleated giant cell showing thick wall (black arrow head) with irregular greyish amorphous granular material inside (x100, GMS-HE), E) ‘adiaspore’ showing retraction of inner wall simulating septation (pseudo septation) (black arrow head) (x100, GMS-HE), F) ‘adiaspore’ showing greyish granular contents adherent to inner wall (black arrow head) (x100, GMS-HE), G,H) ‘adiaspore’ showing degeneration and collapse inside multinucleated giant cell (black arrow heads) (x100, GMS-HE).

Amidst suppuration as well as within the granulomas and giant cells were seen ‘round to oval ‘unicellular structures (Adiaspores) ranging from 25 to 80μm diameter with a thick trilaminated wall ranging from 6- 20 μm wide (Figure 1, Figure 2). The laminated wall showed three distinctive zones with the outer zone densely pink, wider middle zone faintly amphophilic, and inner zone faintly pink (Figure 2B). The majority of these spores were ‘empty’ looking rings. Some of them showed basophilic to amphophilic granular material diffusely as well as in aggregates abutting the inner wall (Figure 1B). Some were degenerated, collapsed, either ingested by multinucleated giant cells or lying freely amidst suppuration or giant cells seen entering into the fragmented large spores (Figure 3F, Figure 4G). Some of the spores showed eosinophilic ‘Splendore Hoeppli’ material around them with adherent neutrophils (Figure 1E). Suppuration showed neutrophils, degenerated nuclear debris, macrophages, lymphocytes, plasma cells and some eosinophils (Figure 1,Figure 2). Some larger irregular ovoid spores looked like broad aseptate non-branching hyphae (Figure 2C, D). Some of the old degenerating spores showed retraction of the inner wall simulating septation (‘pseudo-septation’) (Figure 1C, Figure 2D, Figure 3B, Figure 4E). There was no evidence of ‘budding’ or ‘endosporulation’ or typical ‘septation’.

Special stain periodic Acid Schiff stain showed well-delineated spores with refractile pink trilaminated thick wall with adherent aggregates of pink granular material (Figure 3B, C).

Special stain Gomori’s Methenamine Silver showed well-delineated large spores with brown black thick laminated wall and empty cytoplasm. Some showed faint gray granular material inside the cytoplasm (Figure 4).

Culture was not possible as there was no clinical suspicion and hence tissue received was formalin fixed. The case was diagnosed based on histomorphology as ‘Adiaspiromycosis’.

He was treated with topical Luliconazole for two weeks and oral Fluconazole for 7 days. There was complete healing of the site with no recurrence on follow up for one year.

## Discussion

Adiaspiromycosis is a chronic fungal infection caused by the dimorphic fungi Chrysosporium Parvum var Crescens now called “Emmonsia Crescens” belonging to the genus Emmonsia, order Onygenales, family Ajellomycetes. Other members of the family Ajellomycetes include Blastomyces, Histoplasma, Paracoccidioidomyces, Emergomyces and Emmonsiellopsis. Genus Emmonsia includes E crescens, E parva, E pasteuriana and E Africanus. Only E crescens and E parvum produce ‘adiaspores’ while E pasteuriana and E Africanus produce small budding yeasts but not adiaspores. E crescens produces larger ‘adiaspores’ and are considered responsible for “Adiaspiromycosis”, while E parva produces smaller adiaspores and are reclassified as Blastomyces parva (Blastomyces Spp) (1–6).

The disease is relatively rare in humans where they are accidental hosts. The disease is prevalent amongst rodents and domestic animals (7–10). Humans contract the infection by inhalation or inoculation of spores. Most of the documented cases are localized at the site of entry with the lung being the most involved site (11–14). Rarely the spore disseminates to distant sites, particularly in immunocompromised hosts (15). Other sites of involvement include the bone, nose, paranasal sinuses, eye and heart (16). Primary cutaneous involvement is a rare distinct entity (16–21).

The organisms are soil saphrophytes, and exhibit thermal dimorphism with asexual reproduction often occurring at room temperature where hyphal forms produce ‘conidia’ often directly from hyphae (‘lollipop’ like conidia also called ‘aleurioconidia’). The conidia that are released into the external environment grow and produce hyphae and the cycle continues (3,4,19).

In human tissue, exclusively only spores (‘conidia’) are seen and mycelial forms are rarely seen. They grow in size without multiplication, a unique feature amongst fungi (hence ‘non-replicating’ spores also called ‘Haplospores’ and the disease “Haplomycosis”). With time they enlarge to form large ‘adiaspores’, and elicit a granulomatous response with fibrosis forming characteristic ‘adiaspiroma’. They do not produce hyphae, and hence it is uncommon to see hyphal structures in tissue (1–4,11,15). In human tissue (370C), ‘adiaspores’ lose their biological activity and viability. They enlarge in the direction of less tissue resistance and hence tend to take various shapes including ovoid, ellipsoid, cylindrical, and large ‘aseptate non branching hyphae like’ (Figure 2C, D). They develop distinctive refractile thick walls (20- 30μm wide) that are bilaminate or trilaminate. In Hematoxylin & Eosin-stained sections, the thick chitinous outer wall appears eosinophilic to amphophilic, while the inner wall is irregular and stains faintly eosinophilic. Both PAS and GMS delineate the laminated wall into three distinctive zones as an intensely dark staining outer wall, wider faintly stained middle zone, and moderately stained wider irregular inner wall (1–3).

With time, the nuclei undergo degeneration and disappear or form precipitate or aggregates, and hence fully developed ‘adiaspores’ are ‘empty looking’.’ In H&E-stained sections, the cytoplasmic contents including precipitated nuclear material appear as unstained or faintly stained amorphous granules in the center or may get aggregated at the inner portion of the thick wall adding to the substance of the thick wall. They appear faintly pinkish in PAS stain and faint gray in GMS stain. Sometimes the inner wall retracts from the outer wall creating a large empty space that may look like ‘central septation’ (pseudo-septation) and is likely to be confused with division of the organism (Figure 1C,Figure 2D, Figure 4E). The structure of the inner wall is relatively complex, exhibiting irregular projections (Figure 2) and are ‘porous’ in nature simulating ‘honey combing’ or ‘mesh like’ ultra-structurally. The other differential stain to characterize the distinct zones include Alcian Blue which typically stains the inner wall red and outer wall blue (11).

They elicit a suppurative and granulomatous response with typical epithelioid cells, lymphocytes, and multinucleate giant cells engulfing empty rings of ‘adiaspores’ that may collapse or rupture. These empty rings of ‘adiaspores’ are seen amidst suppuration where they are surrounded by a collar of neutrophils (Figure 3F) or may exhibit an antigen antibody reaction with deposition of ‘Splendore Hoeppli’ material (Figure 1F). Characteristically, necrosis and calcification are absent. Typically, the granulomas are ‘non-necrotic’, and hence calcification is rare and are walled by fibrous tissue, forming characteristic ‘Adiaspiroma’ (1–4,14).

The size of the ‘adiaspore’ will give a clue to the duration of lesion. It may reach sizes of up to 50- 80 μm or more. On average it takes about 6- 8 weeks for spore to reach the size of 100 μm and it may reacha size up to 500 μm in long standing cases. Enlarging adiaspores may compress smaller airways with respiratory compromise (1,11,20,21).

As the lesion gets older, adiaspores tend to degenerate, become brittle and easily fold, fragment, become granular, and some become firm to hard so that during microtomy the entire spore gets dislodged by the microtome knife and hence one may see central empty space in granulomas (1).

## Diagnosis

Histopathology is the mainstay in diagnosis. Most of the cases are diagnosed as an incidental finding (1–3,13). Culture of the organism is only possible from environmental sources and infected animals but not from human clinical material due to non-viability of ‘adiaspores’. It is possible that human body temperature of 370C does not favor not only the reproductive process but also the viability of the organisms. The fact that adiaspores are trapped inside thick-walled granulomas may make them nonviable (13). Hence, human tissue is the dead end of the life cycle for the fungi. The organism is difficult to isolate in culture from human clinical material. The diagnosis of Adiaspiromycosis mainly depends on their characteristic morphology in histopathology (1–4,13,14). There are no well-established skin tests and serological tests to diagnose the disease (3).

Differential diagnosis of Adiaspiromycosis include Blastomycosis, Rhinosporidium Seeberi, Coccidioidomycosis, Mucorales, helminths such as Dirofilaria & Strongyloides and small blood vessels Table 1.

**Table 1:** Differential diagnosis of *Adiaspiromycosis.*

***Blastomycosis***	Have characteristic ‘double refractile wall; Usually show ‘broad based budding’ yeasts in serial/ deeper sections.
***Rhinosporidium Seeberi***	Usually show sporangia with sporangiospores in serial/deeper sections; spores stain homogeneously black in GMS; spores are ‘Carminophilic’
***Coccidioidomycosis***	Usually show sporangia with sporangiospores in serial/deeper sections; GMS stains only the wall of spores.
***Transverse section of Mucorales***	Hyphal walls of Mucorales are thin; usually show nonparallel walled right angled branching hyphae in serial/deeper sections.
***Helminths (Strongyloides, Dirofilaria)***	Have thick outer cuticle; organs are seen inside.
***Small blood vessels***	Have endothelial cell lining; luminal blood cells; smooth muscle cells in the wall.

The empty rings of ‘adiaspores’ are confused for empty spherules of coccidioidomycosis & Rhinosporidium seeberi, small blood vessels, and helminths such as Strongyloides and Dirofilaria.

Degenerating yeasts of B. dermatitidis may look ‘empty’ and confused for smaller forms of ‘adiaspores’. Though degenerating, yeasts of B. dermatitidis retain the ‘double refractile’ nature of their wall. Serial and deeper sections may reveal typical tissue forms including evidence of ‘broad based budding’. When in doubt, Congo red stain is a very useful differentiating stain, where yeasts of B. dermatitidis are ‘Congophilic’ showing bright red stain of the wall (22). In addition, Adiaspores are ‘non replicating’ and show no evidence of ‘budding’. Adiaspores, although having a thick ‘trilaminar’ wall, are not ‘refractile’ and are congo red negative.

Spherules of both Rhinosporidium seeberi & Coccidioidomycosis show relatively thinner wall than those of Adiaspores and show characteristic endosporulation. Endosporulation is characteristically absent in Adiaspiromycosis.

Transverse sections of helminths usually show typical outer cuticle and organs inside in contrast to empty rings of Adiaspores.

Small blood vessels are usually confused for Adiaspores, but the presence of flattened endothelial cells on the inner side with dark blue nuclei and the presence of red blood cells rule out Adiaspores (Figure 1G) (3,14).

Transverse sections of irregular hyphae of ‘Mucorales’ are sometimes confused with ‘Adiaspores’. Mucorales have a thin wall in contrast to the thick wall of Adiaspores. Mucorales usually show folded large aseptate irregular non parallel hyphae in the wall of blood vessels, amidst the necrotic material and usually show right angled branching. With careful detailed examination of serial & deeper sections one may find ‘typical tissue form’. Mucorales and particularly Rhizopus spp may show ‘ectosporulation’ with characteristic vesicles and released spores (conidia).

Retraction of the inner wall of Adiaspore may create’ septae like partition’ (Figure 1C, Figure 4E), simulating septating or multiplying fungal cell. With careful examination of deeper and serial sections, one may see typical non replicating ‘Adiaspores’.

Inner granular contents of Adiaspores may be confused with endospores of rhinosporidiosis and coccidioidomycosis. In ‘Adiaspores’, the inner granular contents are irregular heterogenous, amorphous, less numerous, may be seen abutting the inner membrane, and stain faintly gray in GMS and faintly eosinophilic to amphophilic in PAS. Endospores of Rhinosporidiosis and Coccidioidomycosis are numerous, more or less uniformly round, with a distinct wall and stain black in GMS and pink in PAS. Endospores of Rhinosporidiosis are mucicarmine positive while granular contents of ‘Adiaspores’ are mucicarmine negative (3).

## Conclusion

Adiaspiromycosis is a rare self-limiting fungal infection and primary cutaneous involvement is a rare distinct entity. It is often an incidental finding in histopathology. Human tissue is the dead end of the life cycle for the fungi. The fungus is difficult to isolate from human clinical material because of the unique thermal dimorphism. Histopathology is the mainstay in diagnosis.

## Conflict of Interest

The authors have no conflict of interest.
